# Frequency Domain Electroretinography in Retinitis Pigmentosa versus Normal Eyes

**Published:** 2012-01

**Authors:** Homa Hassan-Karimi, Ebrahim Jafarzadehpur, Bahram Blouri, Hassan Hashemi, Arash Zare Sadeghi, Ali Mirzajani

**Affiliations:** 1 Department of Medical Physics, School of Medicine, Tehran University of Medical Sciences, Tehran, Iran; 2 Department of Optometry, Faculty of Rehabilitation, Tehran University of Medical Sciences, Tehran, Iran; 3 Farabi Eye Hospital, Tehran University of Medical Sciences, Tehran, Iran; 4 Noor Ophthalmology Research Center, Tehran, Iran

**Keywords:** Electroretinogram (ERG), Retinitis Pigmentosa (RP), Fast Fourier Transform (FFT), Fmod

## Abstract

**Purpose:**

To compare electroretinogram (ERG) characteristics in patients with retinitis pigmentosa (RP) and normal subjects using frequency domain analysis.

**Methods:**

Five basic ERG recordings were performed in normal subjects and patients with a clinical diagnosis of RP according to the ISCEV (International Society of Clinical Electrophysiology of Vision) protocol. Frequency domain analysis was performed by MATLAB software. Different frequency domain parameters were compared between the study groups.

**Results:**

Peak frequency (Fmod) of flicker and oscillatory responses in RP patients showed significant (P<0.0001) high pass response as compared to normal controls. Peak frequency (Fmod) of the other responses was not significantly different between the two groups.

**Conclusion:**

In addition to conventional ERG using time domain methods, frequency domain analysis may be useful for diagnosis of RP. Oscillatory and flicker responses may be analyzed in frequency domain. Fast Fourier transform may reveal two distinct high pass responses (shift to higher frequencies) in Fmod. Time and frequency domain analyses may be performed simultaneously with many modern ERG machines and may therefore be recommended in RP patients.

## INTRODUCTION

Retinitis pigmentosa (RP) is a group of hereditary retinal disorders with a prevalence of 1:4000 which affects approximately 1.5 million people worldwide.^1^ RP is characterized by progressive degeneration of rod and cone photoreceptors resulting in night blindness and progressive visual field loss, eventually leading to severe visual impairment.^1^–^4^

Electroretinography (ERG) is a valuable technique for studying retinal function objectively. It is an efficient method for precise diagnosis and follow-up of patients with RP as well as for evaluation of its prognosis. Usual ERG findings in RP include general reduction in the amplitude of five ERG responses (rod, maximum, oscillatory, cone and flicker). Reduction of scotopic rod response is the first ERG sign. Maximum response which reflects rod and cone activity may be affected after pure scotopic responses become abnormal. Visual electrophysiologists usually concentrate on scotopic and mesopic results for early detection of RP. However, follow-up may be another issue; cone, oscillatory and flicker amplitudes should be considered during follow-up. Another finding in time domain ERG analysis is implicit time. RP patients often show prolonged b-wave implicit times. However, in the early stages of the disease, ERG may show normal amplitude and implicit time values, therefore other signs and symptoms may be used for making a diagnosis.^2^–^4^

Many investigators have attempted to reveal possible mechanisms of the disease based on ERG findings.^1^–^9^ Kondo and Sieving^4^ concluded that post-photoreceptor activity plays a major role in generating flicker ERG responses. Speros^8^ suggested that oscillatory potential may originate from negative and inhibitory feedback circuits between amacrine, ganglion and bipolar cells.

The relationship between RP and ERG findings has been studied in many reports.^3^–^11^ It has been shown that temporal dysfunction of the cone system occurs in the early stages of RP.^2^ Falsini^6^ showed that analysis of flicker ERG is useful to characterize cone system dysfunction in RP. Alexander^7^ suggested that high-frequency flicker ERG may provide better assessment of cone photoreceptor integrity in RP as compared to lower frequencies. Speros et al^8^ reviewed the utility of oscillatory potentials in evaluating disease prognosis and reported that oscillatory potentials become further reduced as RP progresses.

Most studies on ERG in RP have focused on time domain ERG and only few have evaluated frequency domain ERG. Fast Fourier transform (FFT), power spectrum and more recent approaches of wavelet analysis may be applied for ERG recordings based on International Society of Clinical Electrophysiology of Vision (ISCEV) guidelines to overcome false positive and negative results of conventional ERG. Frequency analysis is useful for identifying specific frequency changes in ERG components, while FFT and power spectral density allow detailed estimation of frequency distribution and components.^12^ Therefore, ERG signal analysis in frequency domain may establish more accurate criteria for the diagnosis of RP.

## METHODS

This study included 32 eyes with RP and 22 normal eyes. RP patients were referred from an ophthalmologist after performing routine visual and ocular examinations. All RP patients had early or intermediate disease. Normal sex- and age-matched subjects and RP patients underwent ERG recordings. The examiner was masked to the results of previous ocular and visual examinations. ERG recording was performed according to ISCEV standards.^9^,^13^ Five full-field ERG responses (scotopic and photopic) were recorded by the RETI-port device (ROLAND CONSULT, Brandenburg/Germany) at Noor Eye Hospital. Conventional ERG analysis (time domain) was performed by the examiner. Data transfer from the recording machine to the American standard code for information interchange (ASCII) format was accomplished by an independent technician. ASCII formatted data was transported to MATLAB software. Frequency components were derived from Welch power spectral density estimate of responses (MATLAB Signal Processing Toolbox; version 7.8.0.347-R2009). Frequency domain analysis was performed according to F_mod_ analysis. F_mod_ was extracted from the power spectrum of ERG recordings. F_mod_ corresponds to the frequency of maximum amplitude and maximum occurrence. Therefore, F_mod_ indicates the dominant frequency within the power spectrum.

Frequency components of five recorded responses (rod, maximum, oscillatory, cone and flicker) were compared between normal and RP eyes using independent t-test and multivariate analysis statistical method.

## RESULTS

Time domain ERG analysis revealed significantly (P<0.05) decreased amplitudes in all five recordings (rod, maximum, oscillatory, cone and flicker) in all patients as compared to normal eyes. The amplitude of b-waves in rod, maximum and cone responses was also significantly (P<0.001) reduced in RP patients. Oscillatory P_2_ peak and flicker amplitudes showed significant (P<0.001) reduction in RP patients as well.

Average F_mod_ values for ERG responses are shown in table 1. F_mod_ in maximum (rod and cone) and cone responses was not significantly different between RP and normal subjects. However, F_mod_ was significantly (P<0.05) different between the study groups in rod, oscillatory and flicker responses. The average of F_mod_ significantly increased (high pass shift) in RP patients. Mean F_mod_ for rod response, oscillatory potentials (OPs) and flicker responses in RP patients was 5, 12 and 13 Hz, respectively.

**Figure 1.**
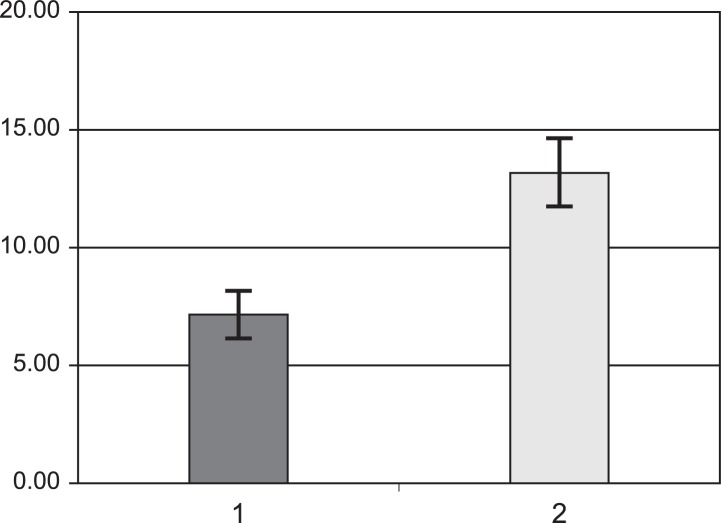
Comparison of F_mod_ for flicker responses in normal subjects (1) and patients with retinitis pigmentosa (2).

**Figure 2.**
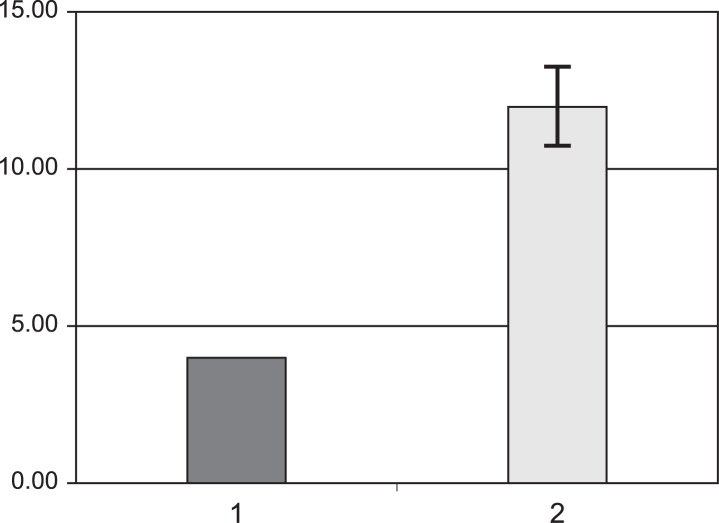
Comparison of F_mod_ for oscillatory potentials in normal subjects (1) and patients with retinitis pigmentosa (2).

Average values of F_mod_ for flicker responses are shown in figure 1, and F_mod_ for OP responses are compared in figure 2.

## DISCUSSION

Frequency stability is an important feature of normal ERG recordings. F_mod_ recordings in all scotopic situations are almost the same in normal eyes (Table 1) indicating that specific cells with certain frequencies of action potentials are activated during normal retinal electrical activity.^14^,^15^ In RP patients however, changes in electrical activity and/or other cellular functions may be responsible for the frequency shift.^16^

Photopic conditions and cone cell activity may be different from scotopic conditions even in normal eyes.^17^ The cone photoreceptor neural network may be totally different from the rod system,^18^ therefore different frequency responses may be seen with cone and flicker responses as compared to rod recordings.

Cones, rods and other cells may show different frequency responses in RP. In the current study frequency responses in RP patients showed an increasing trend in frequency (Table 1). Oscillatory potentials showed more prominent changes in F_mod_. These findings imply that cells and neural circuits are differentially affected in RP (Fig. 2). Cone photoreceptors showed minimal changes in RP, therefore better acuity under photopic conditions is predictable in RP subjects. Night visual impairment is the most common symptom of RP.^1^ In the current study, the flicker response in RP patients was significantly different from normal subjects (Fig. 1). It seems that, despite acceptable cone response in the early stages of the disease, post-synaptic cells show altered frequency responses in RP patients. These results may indicate different post-synaptic neural processing in RP patients.^19^ No similar studies employing frequency domain ERG in RP were found to compare our findings with.

Time domain analysis has shown reduced OP amplitudes in RP subjects.^6^ Speros^8^ described a correlation between circulatory deficiency in the inner nuclear layer of the retina and reduced OPs. Prominent amplitude reduction in flicker responses, in time domain, has been reported by many authors in RP patients.^2^,^3^,^19^ Falsini^6^ believed that the temporal response of the inner and outer retina in RP patients are completely different. This may explain the significant frequency shift we observed in oscillatory and flicker responses of RP patients. Kondo and Sieving^4^ showed that 83% of responses to 30 Hz stimuli in normal subjects are due to post-receptor components and there is also increasing evidence of post-receptor alterations in RP patients.^19^

Many factors may cause a high pass response in frequency domain ERGs, but the inner retinal response may be the most important one in frequency responses.^20^–^22^ High pass shift in RP patients may originate from photoreceptors and/or inner retinal layers. Further studies are required to explain the high pass shift in RP patients with focus on frequency shift.

In summary, combined time and frequency domain analyses may be the best approach for RP patients in clinical practice. This assessment may be useful for differential diagnosis and patient follow-up. Additionally, these findings may fulfill ISCEV demands^9^,^13^ for proposing new analytical methods for ERG and other visual electrophysiological studies.

## Figures and Tables

**Table 1.** F_mod_ electroretinogram components in 32 eyes with retinitis pigmentosa (RP) and 22 normal eyes

**ERG responses**	**Normal**	**RP**	P-value*
Rod	4.0 ± 0.01	5.0 ± 0.24	0.016
Maximum	4.0 ± 0.3	4.7 ± 0.23	0.068
Oscillatory Potentials	4.0 ± 0.02	12.0 ± 0.64	<0.0001
Cone	4.2 ± 0.12	4.3 ± 0.08	0.419
Flicker	7.1 ± 0.48	13.1 ± 0.73	<0.001

* *t-*test
